# A Practical Treatise on the Causes, Symptoms, and Treatment of Spermatorrhœa

**Published:** 1848

**Authors:** 


### Part 2.—Reviews.

### ARTICLE I.

*A Practical Treatise on the Causes, Symptoms, and Treatment of Spermatorrhœa.* By M. LALLEMAND, formerly Professor of Clinical Surgery at the University of Montpelier, Member of the Royal Academy of Medicine of Paris, &c., &c. Translated and Edited by HENRY J. MCDOUGAL, Member of the Royal College of Surgeons of England, &c., &c. pp. 320, 8vo. Philadelphia: Lea & Blanchard. 1848. (From the publishers, and for sale by Joseph Keen, Jr., Chicago.)

A treatise, of the extent of this work, upon the subject of involuntary seminal discharge, may, from the limited notice the subject has received heretofore, seem to our readers to be unnecessary. But, as the numerous affections of the organs of generation, direct and sympathetic, that give rise to debility and irritability, are considered, the work is, perhaps, as brief as is compatible with a full elucidation of the subject. The work before us is an abridgement of the original, which forms three large octavo volumes.

In glancing hastily over this work, we come to the conclusion that spermatorrhœa, as a symptom of disease, if not as a principal affection, is worthy of much more attention than it has heretofore received.

Hypochondriasis and cerebral congestion seem to be very frequent accompaniaments of the affection.

In the cases reported, of which there are sixty-two, we observe that a large proportion seem to have followed excessive sexual indulgence or masturbation, and are accompanied with melancholy.

We have often been struck with the large number of cases of insanity, reported as being caused by masturbation, and conclude that the habit is very frequently as much a result of hypochondriasis as a cause, in which case the excessive irritation to which the organs are subjected not unfrequently gives rise to spermatorrhœa.

The work, altogether, is quite interesting; contains much valuable information; and, where the people are much liable to venereal diseases, or excesses will, doubtless, be of much practical value. We observe that cauterization has been very successful in the hands of the author.

To show the plan of the work, we quote from the introduction:

"It is, however, of great importance to study attentively the symptoms of involuntary spermatic discharges; they are little known, very varied, and capable of stimulating a host of other affections; but their character is independent of the first cause of the disease, and they furnish few indications for the regulation of its treatment.

"On the other hand, the history of this affection is so much in its infancy, that I feel the necessity of proceeding as if I were treating an entirely new subject. I shall, therefore, relate many single cases, before I attempt to arrive at general conclusions. As these cases are very numerous, I must classify them according to some arrangement, and I shall place the CAUSES first in this classification, since they are the most important part of it. Proceeding from the evident to the doubtful, and from the simple to the compound, I shall examine first the causes whose action is most direct and undoubted; and whilst studying the influence of each cause, I shall bring forward the cases in which its action has been energetic, isolated, and when possible, proved by post mortem inspection, and I shall afterwards cite cases in which several causes have acted successively or simultaneously.

"After having examined many cases in this manner, I shall make a general *resume*, in the course of which, I shall comment on whatever relates to the symptoms of the treatment.

"I shall also pay attention to the analogous phenomena which may be observed in the female.

"I propose then to consider this affection of the genital organs in all its varied phases; I shall pass rapidly over what is already known; I shall, on the contrary, insist on the most remarkable errors, and comment fully on all that may seem doubtful or obscure.

"If I were to relate all the cases that have come under my notice, tiresome repetitions would result: I shall, therefore, choose only those which best show the characteristic features of the most important distinctions."

In reference to the pathological anatomy of these organs, our author correctly observes:

"Works on pathological anatomy have hitherto afforded us very little information respecting this important and delicate matter; the omission arises from several circumstances.

"Inflammation of the spermatic organs does not threaten life at its commencement; when the patient dies at an early period of the affection, it is in consequence of some other more serious disease, which engrosses the care of the attendants, so that after death, examination of the spermatic organs is neglected."

Several cases are reported and the post mortem appearances detailed, showing important lesions of structure in the different parts of the genito-urinary apparatus. These are particularly interesting, as showing the result of badly treated cases of gonnorrhœa. The testicles, seminal vesicles, prostate gland, bladder, and kidneys, generally show the most prominent marks of disease.

The following quotation shows the application made of this chapter, to the subject of the work:

"To resume:—All the mucous surface of the genito-urinary organs have the greatest analogy and the most intimate connection with one another. It is by them that inflammation creeps by degrees to the secreting organs of the urine and of the semen. The portion of this membrane which lines the prostate, being in intimate connection with that of the mucous follicles, with that of the ejaculatory ducts, and with that of the bladder—this portion then is the one, the different conditions of which have most effect on all the rest. This connection takes place by means of the lining membrane of the ducts; and is by no means to be considered the result of sympathy, such as exists between the uterus and breasts.

"The excretory canal transmitting the inflammation, must necessarily share its influence. The seminal ducts and vesicles, then, cannot remain unaffected by the action they transmit to the testicles and this is an important consideration when we recollect that these are as much the acting organs in the emission of semen, as the bladder is the organ for the expulsion of urine.

"We shall often find it necessary to apply these facts to the study and treatment of diurnal pollutions, and in passing, it is as well to notice, that the influence of the excretory canals on the secreting organs is not an isolated phenomenon occurring only in the kidneys and testicles, but that it is the result of a general law, applicable to all glands.

"Suction excites the secretion of milk and changes its qualities ; the first drops drawn from the nipple are watery, and the milk afterwards becomes more abundant and better formed in proportion as the suction continues. The introduction of extraneous bodies between the eyelids increases the lachrymal secretion, which sometimes even is so changed, that it irritates and excoriates the skin of the cheeks. The presence of food in the mouth, especially when spiced and savoury, increases the secretion of the salivary glands. During digestion, the liver and pancreas are excited ; and the action of emetics and purgatives produces the same effects. The ejaculatory ducts open on the surface of the prostatic mucous membrane ; is, then, the important part which this membrane plays in the production of spermatorrhœa, a cause for wonder ?"

We next have eight chapters on the causes of spermatorrhœa, constituting the main body of the work. In these, are reported fifty-eight cases, of which we make the following brief summaries. In chapter third there are five cases illustrative of the effects of blennorrhagia, as a cause. In reference to it, the author observes :

"Blennorrhagia is the most active and the most direct, as well as the most easily appreciated, of all these causes, and this is why I have commenced by reporting cases in which it has played a principal part. When these cases are examined separately with some attention, we soon perceive that the discharge has been preceded, accompanied, or followed, by some circumstances capable, by their own action, of giving rise to spermatorrhœa. It is necessary to pay attention to this point."

In chapter fourth there are five cases, showing cutaneous affections to have caused the disease.

Chapter fifth gives fourteen cases, in which it was caused by diseases of the rectum, in most of them, by the irritation of ascarides, the expulsion of which effected a cure; in others, by the pressure of fæces, hemorrhoids, &c., in which removal was followed by recovery.

In chapter sixth, abuse of the organs of generation is shown to cause spermatorrhœa, by the report of seven cases. It might be remarked that masturbation was practiced by a large number of the cases, reported under other heads.

Chapter seventh, gives ten cases of venereal excess, that resulted in the disease.

Chapter eighth speaks of the action of certain medicines, as causing it, with a report of four cases. The medicines are astringents, purgatives, narcoties, cantharides, camphor, nitrate of potassa, ergot, coffee, and tea.

The close of the following paragraph shows how easy it is to give a coloring to facts, if facts they are, to make them suit the author's purpose :

" *Ergot of Rye.*—This singular production seems to act with as much energy on the genital organs of man, as on the female uterus. In the districts where spurred rye is common and the peasantry are not careful to seperate the diseased grain from the healthy, the men show a considerable disposition to commit venereal excesses, and the women frequently abort. The population generally, also present signs of premature decrepitude, which we can easily imagine, may arise from involuntary seminal discharges brought on by the excesses they commit."

Chapter ninth has two cases to show the influence of the cerebro-spinal system of nerves in the production of the disease. We extract the following given by the translator :

" A private in the engineers wishing to get out of his barracks to visit a female, fell from a great height on his buttocks. Serious concussion resulted, but no fracture. Notwithstanding bleeding, leeches, cupping, issues, &c., the lower extremities remained paralysed. After a time, however, galvanism restored slight motion, and obscure sensibility. Still the glans, the prepuce, and skin of the penis and scrotum, remained completely insensible. Pinching, and pins driven into them, were unperceived by the patient. Catheterism, which at first was frequently necessary, never induced complaints. But chronic vesicular catarrh supervening, I cauterized the bladder and its neck, and this operation gave just as much pain as in other patients.

"The same phenomena followed. At first the urine was sanguinolent and thick, but soon lost this appearance, and was passed with greater force and facility. Whilst treating this patient, I often found the penis in complete, and indeed remarkable erection. I mentioned this to the patient, who told me that he often suffered from this state of priapism, which he found very disagreeable on account of the obstacle which it formed to the discharge of urine. In order to relieve himself, he had several times tried masturbation, but had never been able to procure ejaculation, notwithstanding the erection was perfect, and he had persevered in his manœuvres. He experienced no pleasure, and only attempted it in the hope of relieving the priapism. Having one day obtained permission to leave the hospital, he visited the female, to see whom he had scaled the barrack walls in so unfortunate a manner. He passed several hours with her in almost continual connection, without being able to procure ejaculation, and without experiencing the least sensation. On the other hand, all his functions were well performed, with the exception of slight costiveness; he gained flesh daily, and his moral faculties were not affected; abundant nocturnal pollutions took place at long intervals, and were preceded by erotic dreams, but accompanied with but little pleasure.

"This case shows clearly the special influence of the spinal nerves in contra-distinction to that of the branches of the sympathetic, distributed to the different parts of the genital apparatus. In fact, in this patient, all the phenomena dependant on the cerebro-spinal apparatus were abolished, whilst the others had not experienced the least change. Voluntary ejaculation was impossible, because the penis had lost all sensibility, and consequently, all its influence over the seminal vesicles. This confirms what I have already stated respecting the difficulty of ejaculation caused by intoxication or narcotism. It is sufficiently evident, that alcaholic drinks, &c., when the stupor is perfect, may retard ejaculation or even render it impossible, although erection may be complete. In this patient, there was constant and energetic priapism, which was not accompanied by any lascivious ideas, because it was produced directly by the accumulation of semen in its reservoirs, without any sensation being transmitted to the encephalon, at least during the waking state. But during sleep, all the senses being inactive, as well as the cerebro-spinal system, and the nerves derived from it, sensations transmitted by the branches of the trisplanchnic, might awaken images and associations of ideas, as well as produce from time to time lascivious dreams and nocturnal pollutions—proving that these phenomena are directly under the control of the great sympathetic."

And chapter tenth, on congenital predisposition, gives nine cases of malformation of the prepuce, &c., some diseases of the organs, which produced spermatorrhœa.

The remaining chapters of the work, are devoted to the symptoms and treatment of the disease.

The treatment, of course, is varied considerably according to the cause, and the appropriate remedy suited to each, is generally recommended.

"In case of chronic inflammation or irritation of the urethra," cauterization is recommended. This is to be effected by the nitrate of silver applied by means of the author's porte-caustique, which is generally familiar to the profession.

With these brief notes, we leave the work with a sense of gratification, similar to that attending the washing of hands after the performance of a dirty manipulation—conscious of having discharged a duty in the performance of a disgreeable office. E.

